# Author Correction: Coherent diffractive imaging of microtubules using an X-ray laser

**DOI:** 10.1038/s41467-019-12151-3

**Published:** 2019-09-05

**Authors:** Gisela Brändén, Greger Hammarin, Rajiv Harimoorthy, Alexander Johansson, David Arnlund, Erik Malmerberg, Anton Barty, Stefan Tångefjord, Peter Berntsen, Daniel P. DePonte, Carolin Seuring, Thomas A. White, Francesco Stellato, Richard Bean, Kenneth R. Beyerlein, Leonard M. G. Chavas, Holger Fleckenstein, Cornelius Gati, Umesh Ghoshdastider, Lars Gumprecht, Dominik Oberthür, David Popp, Marvin Seibert, Thomas Tilp, Marc Messerschmidt, Garth J. Williams, N. Duane Loh, Henry N. Chapman, Peter Zwart, Mengning Liang, Sébastien Boutet, Robert C. Robinson, Richard Neutze

**Affiliations:** 1 Department of Chemistry and Molecular Biology, University of Gothenburg, Box 462, 40530 Gothenburg, Sweden; 2 Molecular Biophysics and Integrated Bio-Imaging Division, Lawrence Berkeley National Laboratory, 1 Cyclotron Rd, 94720 Berkeley, CA USA; 3 Center for Free-Electron Laser Science, Deutsches Elektronen-Synchrotron DESY, 22607 Hamburg, Germany; 4 Linac Coherent Light Source, SLAC National Accelerator Laboratory, Menlo Park, CA USA; 5 The Hamburg Center for Ultrafast Imaging, 22761 Hamburg, Germany; 6 Institute of Molecular and Cell Biology, Biopolis, A*STAR (Agency for Science, Technology and Research), 138673 Singapore, Singapore; 7 Department of Physics, National University of Singapore, 117551 Singapore, Singapore; 8 Department of Physics, University of Hamburg, 22761 Hamburg, Germany; 9 Department of Biochemistry, National University of Singapore, 117597 Singapore, Singapore; 10 Research Institute for Interdisciplinary Science, Okayama University, Okayama, 700-8530 Japan

**Keywords:** Image processing, SAXS, X-ray crystallography

Correction to: *Nature Communications* 10.1038/s41467-019-10448-x, published online 13 June 2019.

The original version of this Article contained an error in the author affiliations.

Stefan Tångefjord was incorrectly associated with Discovery Biology, AstraZeneca R&D, Pepparredsleden 3, 43189, Mölndal, Sweden, but instead should be affiliated with Department of Chemistry and Molecular Biology, University of Gothenburg, Box 462, 40530, Gothenburg, Sweden.

This has now been corrected in both the PDF and HTML versions of the Article.

