# Supplementary material for: Coherent diffractive imaging of microtubules using an X-ray laser
Source: Nat Commun. 2019 Jun 13;10:2589. doi: 10.1038/s41467-019-10448-x (PMC6565740; doi:10.1038/s41467-019-10448-x)
Supplement: Supplementary file 2 — Supplementary Information [file 41467_2019_10448_MOESM2_ESM.pdf]

SUPPLEMENTARY INFORMATION FOR:

COHERENT DIFFRACTIVE IMAGING OF MICROTUBULES USING AN X-RAY LASER

Gisela Brändén, Greger Hammarin, Rajiv Harimoorthy, Alexander Johansson, David Arnlund, Erik Malmerberg, Anton Barty, Stefan Tångefjord, Peter Berntsen, Daniel P. DePonte, Carolin Seuring, Thomas A. White, Francesco Stellato, Richard Bean, Kenneth R. Beyerlein, Leonard M. G. Chavas, Holger Fleckenstein, Cornelius Gati, Umesh Ghoshdastider, Lars Gumprecht, Dominik Oberthür, David Popp, Marvin Seibert, Thomas Tilp, Marc Messerschmidt, Garth J. Williams, N. Duane Loh, Henry N. Chapman, Peter Zwart, Mengning Liang, Sébastien Boutet, Robert C. Robinson, Richard Neutze

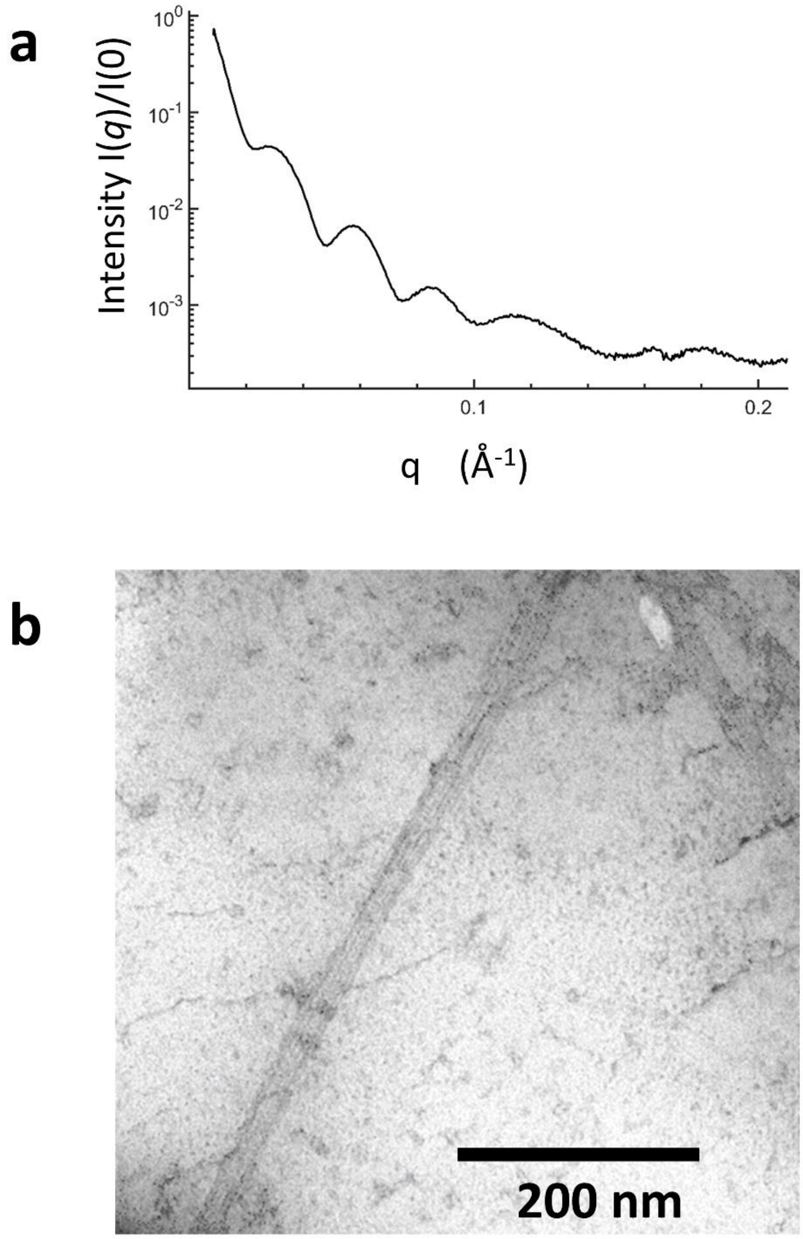

Supplementary Figure 1: Characterization of microtubules. (**a**) Small-angle X-ray scattering data of microtubules collected at 17 °C using X-ray exposures of 2 seconds. Samples were prepared at a concentration of 90 µM at 37 °C and stabilized by the addition of 10 µM taxol. Normalized intensities are plotted on a logarithmic scale. (**b**) Negative-stain electron micrograph of microtubules. Samples were prepared at a concentration of 18 µM at 37 °C and were stabilized by glutaraldehyde.

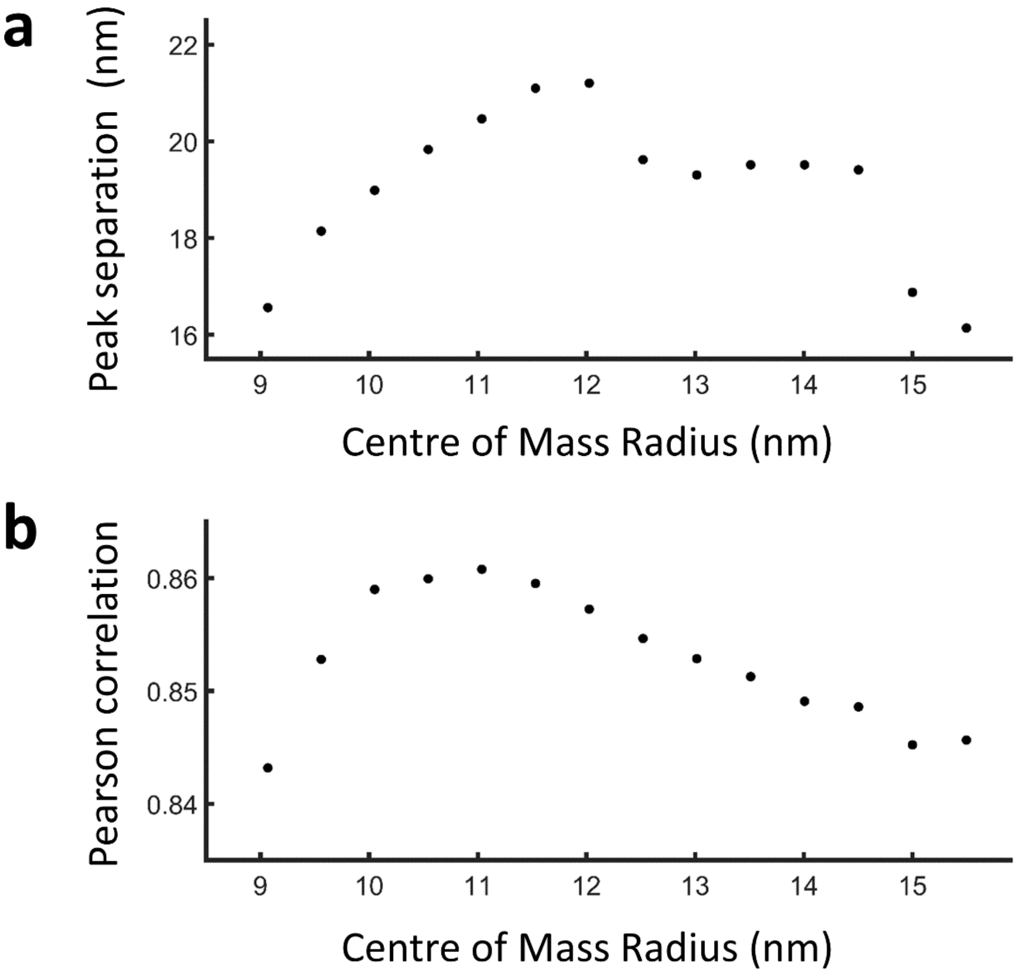

Supplementary Figure 2: Control iterative phase retrieval calculations. (**a**) Peak-to-peak separation for the maximum intensities recovered in the real-space 2D projection image after phase retrieval reconstructions using a variety of featureless tubes for the initial phases (Fig. 5D). (**b**) Pearson correlation scores recovered between the equatorial experimental intensities (central left panel of Fig. 2C) and the Fourier Transform of the final 2D projection image after phase retrieval (Fig. 5D). The center of mass radii (COM) of the starting model are calculated from the inner and outer diameter of the featureless tubes used for initial phases. For example the COM radius calculated for a featureless tube approximating a 13-3 pf microtubule (pdb entry 5SYF[13]) is 11.2 nm. Relative changes in the COM radius for other microtubule forms can be estimated from Table 1 of reference[48].
